# 

**Published:** 1892-11-19

**Authors:** 


**Notices of Births, Deaths, and Marriages,** are inserted in this column at a charge of 2s. 6d. for an announcement not exceeding four lines.

### NOTICE TO CORRESPONDENTS.

All MS., Letters, Books for Review, and other matters intended for the Editor should be addressed The Editor, The Lodge, Porchester Square, London, W.

The Editor cannot undertake to return rejected M.S., even when accompanied by stamped directed envelope.

**Notice to Advertisers and Subscribers.**

All Advertisements, Orders for Copies of The Hospital, and Business Communications should be addressed to The Publisher, *not to the Editor*, The Hospital, 140, Strand, W.C.

The Cost of Subscription, post free, is as follows:—Per week, 2½d.; per quarter, 2s. 6d.; per half-year, 5s.; per annum, 8s. 6d. Foreign:—Per annum, 10s. 8d.; payable, in all cases, in advance.

**"Burdett's Hospital Annual," 1891—1892.**

Third year of publication. 600 pp. Boards, gilt lettering. A most complete guide to British and Colonial Hospitals, Dispensaries, Nursing and Convalescent Institutions, and Asylums. Price 3s. 6d. Published by The Scientific Press (Limited), 140, Strand, W.C.

**NOTICE.—The Index for Vol. XII. (ending September, 1892) is on sale at the Office of this Journal, price 2½d., post free.**

### Scraps and Gleanings.

A new roof has been added to the Hereford General Infirmary at a cost of £250.

Princess Christian has accepted the presidency of the Norwood centre of the St. John Ambulance Association.

Mr. W. Glaholm Black, F.R.C.S., M.R.C.S., has been appointed honorary assistant surgeon to the Newcastle Infirmary.

The Hospital Sunday Collections in Birmingham this year in aid of the General Hospital amounted to between £4,000 and £5,000.

The Comte and Comtesse de Paris have forwarded 12 brace of pheasants to the French Hospital in London, for the use of patients in that institution:

A sum of £16,200. collected in offices and workshops, has been received at the central offices of the Hospital Saturday Fund on behalf of the fund for the present year.

Surgeon Major General F. W. de Fabeck, of the Indian Medical Department, has received, on the recommendation of the Indian Government, a good service pension.

The Strolling Players have promised to act "The Old Love and the New" in aid of the Surrey Convalescent Home, Seaford, at St. George's Hall, Langham Place, on Tuesday, November 29th.

The Town Council of Lymington propose to erect by voluntary contributions an infectious hospital, which will be open to all the parishes in the union. It is estimated that the building will cost £1,000.

Princess Beatrice, President of No. 3 District, Metropolitan Centre, St John Ambulance Association, has presented an autograph photograph of her Royal Highness to Mr. C. Alan Palmer, Honorary District Secretary.

The forty-eighth annual report of the Dudley Dispensary shows that 30,654 patients were treated at the dispensary, the number of visits paid by the resident medical officer, 3,520; and 1,670 cases were supplied with surgical appliances.

The Commissioners of the Clapham Public Library have decided to open their reading and magazine rooms on Sundays from three to nine o'clock, commencing with the first Sunday in December, and ending with the last Sunday in May, 1893.

Fleet-Surgeon Woods has been giving a course of nursing lectures at Balmoral to an audience of over 20 people of the Royal estate and household. Princess Henry of Battenberg and all other ladies attending go through a bandaging practice besides.

The working-men's committee of the Coventry and District Hospital Saturday Fund for 1892 have handed over £1,274 18s. 11d. to the Coventry and Warwickshire Hospital. The total collection amounted to £1,313 10s. 11d., and the expenses to £38 12s.

The Duke of Portland recently remitted £150 to the treasurer of the Nottingham General Hospital, being part of the fees paid by visitors to Welbeck Abbey during the past summer. The collections on Hospital Sunday in aid of the institution amounted to £263 9s. 10d.

Mr. W. Liddon, who for the past 33 years has held the office of surgeon to the Taunton and Somerset Hospital, has, owing to ill-health, been obliged to resign. At the annual meeting of the Hospital it was proposed to add a children's ward and other necessary requirements.

Mrs. Janet Wills, widow of Mr. W. H. Wills, one of the creators of *Punch*, has bequeathed £1,000 to the Newspaper Press Fund. Mr. Wills was a great friend of Charles Dickens; he was also a founder, and for some years before his death in 1880, vice-president of the Newspaper Press Fund.

Mr. F. A. Bevan, presiding over the half-yearly meeting of the British Home for Incurables, announced that another £150 had been received from the Princess of Wales, making in all £350 from the proceeds of the sermon preached by Canon Fleming on the death of the Duke of Clarence.

The Prince of Wales will lay the first stone of the new wing of the St. Mary's Hospital, Paddington, which is to be named after the late Duke of Clarence, on December 17th and not on December 14th as previously announced. The Princess of Wales is unable to take part in the ceremony, and it is not known whether the Duke of York will be present.

The Triennial Warren Prize, value 500 dollars, given by the General Hospital, Massachusetts, U.S., for the best treatise on "Rickets," has been awarded to Mr. John Strahan, M.D., Belfast. In 1886 Dr. Strahan gained the Fothergill Gold Medal of the London Medical Society for an essay on "Typhoid Fever."

The Governors of the Derbyshire Infirmary in celebrating their eighty-third anniversary, expressed their sincere regret at the loss of the three friends, who had during their lifetime done so much for the institution namely, the late Duke of Devonshire, Canon Oliver, and the President, Sir William Owen.

The Vice-Presidents and Committee of the Birmingham General Hospital have presented Sir John Jaffray with an illuminated address as a memento of his long connection with charity as a member of the board, as the founder of the Auxiliary Suburban Hospital, and as chairman of the Birmingham Musical Festival.

The Royal Humane Society's medal and certificate have been presented to James Willoughby Jardine, a student at Eton College, for saving his cousin from drowning in North Devon. The presentation was made by the Rev. Dr. Warre, head master, in the college quadrangle, in presence of the whole school.

Sir Stafford Northcote, M.P., has been elected president for the ensuing year of the West of England Eye Infirmary. During the past year 2,301 patients have been under treatment in the hospital; of this number 1,972 have been discharged, leaving 282 out patients and 47 in-patients under treatment on September 3rd.

The foundation stone of the New Southport Infirmary was laid by the Mayor, Alderman Dr. Pilkington, J.P.C.A., in the presence of not less than 6,000 spectators. During the afternoon all members of the friendly societies who had taken part in the procession enjoyed a substantial tea which had been prepared for them. Later in the evening the Cyclists' Parade and torchlight procession took place, and the day ended with a public ball in aid of the Infirmary, at which 270 guests were present.

### THE STUDENT OF MEDICINE.

[All communications for this Supplement should be addressed to The Editor of The Hospital, at 140, Strand, with the words, "Students' Column," written in the left hand top corner of the envelope.]

### VACANCIES.

The following vacancies are announced this week, the amount of salary in each case being given after the name of the institution:

**Resident Medical Officers.**

Birmingham General Dispensary, Resident Surgeon, doubly qualified—Salary £150 per annum, and £30 per annum allowance for cab hire. Applications to the Secretary by November 16th.

Brighton, Hove, and Preston Dispensary, House Surgeon to the Western Branch, unmarried, doubly qualified—Salary £140 per annum (less board at 8s. per week), with furnished apartments, coal, gas, and attendance. Applications to the Assistant Secretary by December 15th.

Cancer Hospital (Free), Fulham Road, S.W., House Surgeon. Appointment for six months—Salary at the rate of £50 per annum, with board and residence. Applications to the Secretary by November 18th.

City of London Hospital for Diseases of the Chest, Victoria Park, E., House Physician—Board, residence, and allowance for washing provided. Appointment for six months. Applications to the Secretary at the Office, 24, Finsbury Circus, E.C., by December 8th.

East London Hospital for Children, Glamis Road, Shadwell, E., House Physician; board and lodging provided. Applications to the Secretary by November 16th.

General Hospital, Barbadoes, West Indies, Junior House Surgeon—Salary £200 per annum, and quarters. Appointment for three years. Applications to the Secretary by December 1st.

Hospital for Sick Children, Great Ormond Street, W.C., House Surgeon, unmarried. Appointment for six months—Salary £25, with board and residence. Applications to the Secretary by November 15th.

Liverpool Northern Hospital, Assistant House-Surgeon, doubly qualified—Salary £70 per annum, with residence and maintenance in the house. Applications to the Chairman of the Committee by November 23rd.

Northern Infirmary, Inverness, House-Surgeon and Dispenser—Salary £75 per annum, with board, &c. Applications to the Honorary Secretary, Mr. Duncan Shaw, W.S., by November 26th.

Wolverhampton Eye Infirmary, House Surgeon—Salary £60 per annum, with board, rooms, and washing. Applications to the Secretary by November 22rd.

Monkstown Hospital, County Dublin, Resident Medical Officer—Salary £40 per annum, with board, apartments, gas, and coal. Applications to the Honorary Secretary by November 14th. Election on December 1st.

New London County Asylum, Claybury, Woodford, Essex, Medical Superintendent, doubly qualified—Salary £1,000 per annum, with house, coals, lighting, milk, and vegetables. Applications on form to be obtained of Mr. R. W. Partridge, Clerk to the Asylum Committee, 21, Whitehall Place, S.W., by November 25th.

Paddington Infirmary, Assistant to the Medical Superintendent and Assistant Medical Officer of the Workhouse; unmarried; age between 23 and 30—Salary £100 per annum, rising £10 annually to £120, with board, lodging, and washing. Applications to H. F. Aveling, Clerk to the Guardians, 289, Harrow Road, by November 12th.

Royal Hospital for Incurables, Dublin, Resident Medical Officer, qualified in Medicine, Surgery, and to compound medicines—Salary £70 per annum, with board and furnished apartments. Appointment tenable for two years. Applications to Mr. Thomas Grey, Registrar. Election on November 14th.

Royal Infirmary of Edinburgh, Superintendent, must be member of medical profession—Salary £500 per annum, with free house and gas. Applications to Mr. James S. Trainer, Treasurer and Clerk, by December 5th.

St. Luke's Hospital, London, E.C., Clinical Assistant; appointment for six months. Board and lodging. Applications to the Secretary by November 24th.

Sheffield General Infirmary, House-Surgeon, doubly qualified—Salary £120 per annum, with board, lodging, and washing, with a prospective advance of £10 per year for the second and third years. Applications to "Medical Staff," to the care of the Secretary, by November 19th.

Sheffield General Infirmary, Assistant House Surgeon, doubly qualified—Salary £80 per annum, with board, lodging, and washing. Applications to the "Medical Staff," to the care of the Secretary, by November 19th.

West London Hospital, Hammersmith Road, W., House Physician. Appointment for six months. Board and lodging provided. Applications to R. J. Gilbert, Secretary, by December 7th.

West London Hospital, Hammersmith Road, W., House Surgeon. Appointment for six months. Board and lodging provided. Applications to R. J. Gilbert, Secretary, by December 7th.

### THE STUDENT OF MEDICINE—(*continued*).

### Non-Resident Medical Officers.

Brompton and Knightsbridge Provident Dispensary, 28, Fulham Road, S.W., Medical Officer. Applications to Dr. Bland, 33, Rosary Gardens, by November 15th.

Dental Hospital of London and London School of Dental Surgery, Leicester Square, Demonstrator—Honorarium £50 per annum. Applications to Morton Smale, Dean, by November 21st.

Dental Hospital of London, and London School of Dental Surgery, Leicester Square, Lecturer on Dental Metallurgy. Applications to Morten Smale, Dean, by November 21st.

Glamorgan County Council, County Medical Officer—Salary £750, of which £150 is intended to cover all travelling, office, and laboratory expenses. Applications to T. Mansel Franklin, Clerk, Glamorgan County Offices, Cardiff, by November 21st.

Hospital for Sick Children, Great Ormond Street, W.C., Assistant Physician. Applications to the Secretary by November 15th.

Metropolitan Hospital, Kingsland Road, N.E., Assistant Surgeon; must be F.R.C.S.Eng. Applications to Charles H. Byers, Secretary, by November 14th.

Hospital for Sick Children; Great Ormond Street, W.C., Ophthalmic Surgeon. Applications to the Secretary, by November 15th.

Metropolitan Hospital, Kingsland Road, N.E., Assistant Physician. Applications to Charles H. Byers, Secretary by November 28th.

New Hospital for Women, Euston Road, Lady as Assistant Dispenser—Salary £25 per annum, rising to £35. Applications to M. M. Bagster, Secretary, by November 23rd.

Royal College of Surgeons of England.—Election to the Court of Examiners. Applications to the Secretary by November 30th.

Royal College of Surgeons of England, Examiner in Anatomy for the Second Professional Examination. Applications to the Secretary by November 24th.

St. Mungo's College, Glasgow. Chair of Anatomy. Applications to Henry Lamond, Secretary, by December 1st.

Taunton and Somerset Hospital, Honorary Surgeon. Applications to J. H. Biddulph Pinchard, Secretary, 13, Hammet Street, Taunton, by November 21st.

Westminster Hospital, Broad Sanctuary, S.W., Second Dental Surgeon. Applications to S. M. Quennell, Secretary, by November 15th.

Resident Medical Superintendent.

